# Transactions of the American Medical Association, Vol. VII.

**Published:** 1855-03

**Authors:** 


*Transactions of the American Medical Association.* Vol. VII.

In our last number we noticed the report of the Committee on Medical Education. The next paper is the report of the Committee on the Epidemics of Kentucky and Tennessee. We have only time to examine that part of the report devoted to typhoid fever. In reference to the cause of the disease, Dr. Grant of Memphis says: "Every year's observations but tend to confirm the conviction that the derangements of the *innervation* so palpably prominent among the symptoms characterizing the disease are produced by an unknown *poison* acting on the *nervous system*, through the agency of the *blood;* and that this poison is not the same that originates the intermittent and remittent forms of fever, as is proven by differences in symptoms and duration, and especially by the failure of an universally acknowledged remedy for the *one* to prove a curative agent for the *other.*"

Dr. McNelly, of Fayetteville, Tenn., says: "No one in this country saw typhoid fever, and remittent or intermittent fever in the same family at the same time. I have never seen a typhoid fever change into a bilious fever, or *vice versa.* I have conversed with other physicians of this county, who say the same thing."

Dr. Watson, of Hart Co., Ky., says all his cases occurred in ill-constructed and badly ventilated houses. Dr. Barbour, of Falmouth, Ky., says that "chills are much less apt to follow typhoid fever than bilious fever, thereby leaving the legitimate inference that chills do sometimes follow typhoid fever.

It would seem strange that there should be so much conflicting opinions about a disease that has been observed so long and so carefully as has typhoid fever. The truth, we think, is this: the specific disease is modified in symptoms and pathology by the prevailing endemic and epidemic influences. In this city we have seen typhoid fever followed by chills, which were readily controlled by quinine. On the other hand, we have seen cases with distinct exacerbations of fever, in which quinine was of no use whatever. We could not, however, base a diagnosis on this fact.

In reference to the value of quinine in typhoid fever a great discrepancy of opinion exists.

"While Evans, Chambers, and Sutton, of Ky., and Lenoir, of Tenn., recommended its use in the Report made in 1852, and Dille and Swaine, of Ky., recommend it in that of 1853, Hawkins does not mention it, and Grant unequivocally condemns it. Drs. Bradford and Hart do not mention it. It is true that Hart speaks of tonics being serviceable in the latter stages of the disease; and, by implication, may be understood as in some degree favorable to the use of quinia in such states.

"But Drs. McNelley and Grant, of Tenn., and Walton, of Ky., have nothing to say in its favor. Such, perhaps, is a fair representation of the discordant views of the profession in this section of the country as to the use of quinine. In a neighboring village in Scott County, of two gentlemen, partners in practice, one looks upon quinine as little better than poison in typhoid fever, while the other esteems it as emphatically *the* remedy. Where there is such discrepancy of opinion among so many gentlemen of observation and experience, it must be admitted that there is much difficulty in determining the cases in which quinia is beneficial. For it must be allowed that it is sometimes serviceable, or else there is great error in diagnosis. In consideration of the inherent difficulty in arriving at a correct conclusion, we may be allowed to say a few words as to the reasons for its use, and the objections to it. Those who use it do so on different considerations; mainly as an anti-periodic, and as a remedy for irritability, or, as it is frequently called, *nervousness*. That the cold stage, or stage of depression, is as well marked in typhoid fever as in intermittent or remittent fever, is by no means pretended; nay, that it is usually more or less apt to escape observation is fully admitted. But that such cases do occur is certainly true. Take, for example, the following note, at p. 104 of a Treatise on Typhoid Fever, by W. L. Sutton, M.D., of Georgetown, Ky.: 'I am inclined to think that there is frequently a periodical depression in this disease, which escapes observation.—*Ex. g.* While sponging a patient who complained of warmth, and whose forehead felt warm to my hand, I observed a very perceptible *lividity of the lips and of the alæ nasi;* this lividity, indeed, was *perceptible* in the countenance, but would perhaps have escaped observation had it not been for the nose and lips. The toes were very slightly cool. This was all the evidence of depression. Acting upon this hint, I gave ten grains of quinine a few hours afterwards, and repeated it twice a day for several days, with apparently the most unequivocally good effects.'

"These effects were a free, warm perspiration, which lasted about thirty-six hours, an increase of volume in the pulse, and, apparently, the commencement of convalescence.

"The next question is, was this a case of typhoid fever, or was it an obscure case of bilious fever? In answer, I have to say, that the patient was my own son, his case both tedious and bad, in my own house, and of course I was with him myself almost uninterruptedly. I was aided by my professional friends, who, I take the liberty of saying, are worthy to rank with any in the land; and none of us doubted its being a genuine case of typhoid fever. To the usual symptoms of the disease was added the rose spots, which have rarely been observed here, and free hœmorrhage from the bowels."

Dr. Sutton relates several other cases in which quinine was beneficial. Dr. Grant uses it to equalize the circulation, and remove nervous irritation, and says that if he has ever seen any remedy produce an unequivocally good effect in this fever, it is quinine. He has also seen it do harm. Dr. Welton objects to quinine that it increases the frequency of the pulse—sometimes as much as twenty or thirty beats to the minute in the twenty-four hours.

The next paper is on Erysipelas, by R. S. Holmes, M.D., of St. Louis, Mo.

In reference to the treatment of this disease, Dr. H. remarks:

"We are convinced that the proper treatment in all cases, high and low, is that directed to the augmentation of force in the "dynamic," in the nerve centers; and we are not surprised that the popular curative agent is now iron,—in fact, we are pleased that it is so. It is impossible that any agent can go beyond quinine, in our experience. We assert, after a considerable experience in both diseases, that quinine does not cure intermittent fever with greater certainty than it cures erysipelas. There is but one proviso, and that is, that it is given in a sufficiently large dose. The short-sighted timidity of many physicians is a bar to their success.

We do not give opium as a gentle anodyne, in the same doses that we give it in a mania-a-potu; and we may be satisfied that erysipelas is a disease essentially of a high action and stimulus, requiring a proportional amount of any curative agent. If we have not experience enough, or confidence enough in ourselves, to give quinine in greater doses at one time than six to ten grains, then we had better not try it; it is doing injustice to a faithful agent, to treat erysipelas with quinine if you tremble at a twenty-grain dose of it, or if you are in the habit of treating intermittent fever by "a grain and a half every two hours." Doses like these in erysipelas will only increase the disease; they will heighten the stimulus, when that is the thing you wish to lessen, and at the same time will give no additional power to nerve force. The horror with which some physicians look on the administration of large doses of quinine would be merely absurd, were it not that this is negatively the cause of many deaths, or of long and annoying suffering. Erysipelas is never successfully treated by quinine unless the dose at one time, and repeated according to the occasion, is from ten to thirty grains. If the physician does not know enough about the drug to have the proper confidence in giving this, then he had better resort to tartar emetic, cathartics, iron, etc. These will be more in accordance with his own mental constitution, and not at variance, probably, with that of the patient."

Physicians at the North and East will be surprised at this wholesale administration of the remedy, while those at the South and West will, in most instances, assent to it as judicious. We have been in the habit of using quinine in erysipelas, and have seen it used by others, both in hospital and private practice, but never in doses as large as twenty grains. We do not think it necessary, especially if opium in full doses be combined with it. The Dr. regards quinine as an antidote to the erysipelatous poison He says: "We have seen the disease really snuffed out by the application of a large dose of this remedy."

It seems to us that, granting the truth of Dr. H.'s pathology, namely, that the disease is produced by retained excretions, transformed into poisons in the system, we have two methods of treating the disease, the choice of which should be regulated by our estimate of the activity of the poison and the powers of our patient to resist its action. If the strength be sound, and the vital forces active, it may be sufficient to stimulate the excretions. If, on the contrary, the vitality of the system is low, we may use, either alone or conjointly with the preceding class of remedies, stimulants and tonics. The first, according to our observation, are more generally applicable in the earlier, while the second are more frequently demanded in the later stages of the disease. Dr. H. recommends tartarized antimony as the most powerful eliminating agent in the Materia Medica. For our own part, we should not dare to use it in erysipelas. J.

